# On the role of choline in natural DNA transformation in *Streptococcus pneumoniae*

**DOI:** 10.3389/fmicb.2026.1823130

**Published:** 2026-07-09

**Authors:** Flora Peillard-Fiorente, Chantal Godin, Hélène Gingras, Joanna Lecka, Philippe Leprohon, Marc Ouellette

**Affiliations:** Centre de Recherche en Infectiologie, Axe Maladies Infectieuses et Immunitaires, CHU de Québec, Département de Microbiologie, Infectiologie et Immunologie, Faculté de Médecine, Université Laval, Québec, QC, Canada

**Keywords:** biofilm, capsule gene expression, choline, competence, DNA transformation, *Streptococcus pneumoniae*

## Abstract

**Background:**

Under laboratory conditions, DNA transformation of *Streptococcus pneumoniae* is facilitated by the addition of the Competence Stimulating Peptide (CSP). We recently showed that CSP can be dispensable for natural transformation but this depends on both the strains and the growth medium. Indeed, *S. pneumoniae* D39 biofilms or planktonic cells can naturally transform when grown in CDM medium but not in C+Y medium.

**Methods and results:**

By comparing the recipes of the two media, we supplemented C+Y with a number of metabolites found in CDM and observed that increasing choline concentration in C+Y was sufficient to support natural transformation in D39 but also in *S. pneumoniae* ATCC49619. The addition of choline had a major impact in modulating the D39 transcriptome including in pathways related to competence induction and in the decrease expression of the capsule operon.

**Conclusion:**

A change in the concentration of choline can impact natural transformation and thus contribute to the evolution of *S. pneumoniae*.

## 1 Introduction

*Streptococcus pneumoniae*, a pathogen known for its ability to colonize the human nasopharynx asymptomatically (Bogaert et al., 2004), can also be the cause of severe infections such as otitis media, septicemia and meningitis (Faden et al., 1997; Weiser et al., 2018). This pathogen has the capacity for natural DNA transformation, enabling the acquisition of exogenous DNA and rapid adaptation to changing environments. This adaptability is particularly prominent during nasopharyngeal colonization or biofilm formation within the host mucosa (Domenech et al., 2012), providing an environment conducive to the exchange of genetic information (Marks et al., 2012a). Clinical strains of *S. pneumoniae* have shown an increase in antibiotic-resistant genes during nasopharyngeal carriage (Chi et al., 2007; Manenzhe et al., 2019; Regev-Yochay et al., 2004).

Competence, a collective behavior driven by environmental factors, is central to DNA transformation. Under environmental stress, competence is triggered, leading to genetic transformation and adaptation in *S. pneumoniae* (Claverys et al., 2006; Fontaine et al., 2015). The Competence Stimulating Peptide (CSP) is a critical element for the development of the competence state (Havarstein et al., 1995). Pneumococcus competence for DNA transformation is a well-studied quorum sensing [reviewed in Salvadori et al. (2019)] induced by a two-component quorum sensing system. Competence in planktonic pneumococci is a transient physiological event, heavily dependent on the growth phase, cell density, metabolic activities, and nutritional stresses. Under laboratory conditions, it is greatly enhanced by the artificial supplementation of CSP. Clinical encapsulated strains do not naturally exhibit competence under planktonic conditions (Marks et al., 2012b) but pneumococcal biofilms provide an ideal setting for enhanced genetic exchange, both *in vitro* and *in vivo* (Marks et al., 2012a; Michaelis and Grohmann, 2023). The lysis of bacterial cells within biofilms results in a high concentration of exogenous DNA embedded in the biofilm’s extracellular matrix where the close proximity of competent and non-competent cells in biofilms promotes DNA transformation (Cowley et al., 2018).

Recently we have found that under specific conditions the addition of CSP is not necessary for natural DNA transformation of planktonic *S. pneumoniae* (Peillard-Fiorente et al., 2025). Both the media and the bacterial strains impact this increased natural transformation. For example, the widely studied *S. pneumoniae* D39 strain can naturally transform in the CDM medium but not in C+Y, Columbia or BHI media (Peillard-Fiorente et al., 2025). In contrast to the nutrient rich media BHI and Columbia, the C+Y and CDM media are more defined. This has allowed the comparison of the chemical composition of two defined or semi defined media (C+Y and CDM) and identified choline as a key component modulating natural transformation. Choline nutritional requirements as a component of *S. pneumoniae* cell wall is well-established (Rane and Subbarow, 1940). It is incorporated into teichoic (TA) (Brundish and Baddiley, 1968) and lipoteichoic (LTA) acids (Fischer, 2000), which are essential components of the bacterial cell wall. Early literature has indicated that choline is necessary to achieve competence (Tomasz, 1968). Using a transcriptomic approach, we demonstrated that choline supplementation modulates extensively gene expression and provide clues, such as decreased expression of the capsule operon or increased expression of selected competence operons, on how it can contribute to increased natural transformation.

## 2 Materials and methods

### 2.1 Bacterial strains and growth conditions

Bacteria were grown at 35 °C with 5% CO_2_ on Trypticase soy agar with 5% sheep blood (TSAII, Becton Dickinson) or in C+Y medium (Tomasz and Hotchkiss, 1964) containing 0.004 or 0.804 g/L (rounded up at 0.8 g/L throughout the text) of choline chloride (SIGMA). All strains were preserved at −80 °C in brain heart infusion broth (BHI, Becton Dickinson) containing 15% glycerol.

### 2.2 Static biofilm model

*S. pneumoniae* D39 and derivatives were cultivated in C+Y medium (containing either 0.004 or 0.8 g/L choline chloride) until reaching an OD_600_ equivalent to 4.10^7^ CFU/mL, as determined by CFU counts. The inoculation of sterile 24-well plates, their incubation and the harvest of biofilms was done as previously described (Peillard-Fiorente et al., 2025). The number of viable CFU per mL collected from biofilms was calculated by plating on TSAII agar. The quality of the biofilm was assessed as previously described (Peillard-Fiorente et al., 2025).

### 2.3 Natural transformation efficiency assays

Cell lysates were prepared from *S. pneumoniae* D39-DHFR*^I100L^* (Gingras et al., 2019, 2023) cultures grown to an OD_600_ of 0.4 and then heated at 95 °C for 5 min. The absence of viable cells in the lysate was confirmed by plating on TSAII (Marks et al., 2012b). For biofilm natural transformation, *S. pneumoniae* D39 and its derivatives were grown in C+Y (with choline at 0.004 g/L or supplemented at 0.8 g/L) for 24 h at 35 °C under a 5% CO_2_ atmosphere. The culture supernatant was removed carefully and replaced with a 1:25 mixture of *S. pneumoniae* D39-DHFR*^I100L^*. The biofilms were then incubated at 35 °C in 5% CO_2_ for 72 h. The biofilms were harvested and plated on TSAII to determine the total CFU/biofilm and on CAT agar with 5% (vol/vol) sheep blood supplemented with TMP (8 μg/mL) to assess the number of transformants. For planktonic natural transformation, *S. pneumoniae* was grown at 35 °C under a 5% CO_2_ atmosphere until the OD_600_ reached an equivalent of 4.10^7^ CFU/mL, as determined by CFU counts. At this point, 1 mL of the culture was diluted 1:2 with 1 mL of fresh medium, seeded in a 24-well plate and supplemented with 1:25 lysate of *S. pneumoniae* D39-DHFR*^I100L^*. The cultures were grown for 4 h, prior to biofilm formation (Moscoso et al., 2006), and plated on TSAII to calculate the total CFU. Additionally, CAT agar plates containing TMP (8 μg/mL) were used to assess the number of transformants. The transformation efficiency was calculated as the number of transformants obtained over the total number of pneumococcal cells recovered.

### 2.4 RNA sequencing

*S. pneumoniae* D39 biofilms were grown in two independent biological replicates C+Y medium with a choline chloride concentration of 0.004 g/L or 0.8 g/L for 72 h at 35 °C under a 5% CO_2_ atmosphere. Biofilms were treated with RNAprotect (Qiagen) and total RNA was isolated using RNeasy PowerBiofilm Kit (Qiagen) according to manufacturer’s instructions and as described previously (Lupien et al., 2015). RNAs were treated with DNaseI (Ambion) to ensure the removal of any DNA contamination. Quantification of RNA was performed using a 2100 BioAnalyzer RNA6000 Nano chips (Agilent). For ribosomal RNA depletion, 1 μg of total RNA was treated with Ribo-Zero TM Magnetic Kit for Gram-positive Bacteria (Epicentre). The rRNA-depleted samples were then purified using the RNeasy MinElute Cleanup kit (Qiagen). To create the RNA-seq libraries, 50 ng of the rRNA-depleted RNA was used with the ScriptSeqTM v2 RNA-Seq Library Preparation Kit (Epicentre). The quality of the resulting libraries was analyzed using 2100 BioAnalyzer High Sensitivity DNA Chips (Agilent) and quantified by QuantiFluor. The librairies were sequenced on an Illumina HiSeq system using a 101 bp paired ends reads protocol.

### 2.5 RNA-seq data analysis

The *S. pneumoniae* D39V genome (NCBI accession number CP027540) was used as a reference (Slager et al., 2018) to which the sequencing reads from each sample were mapped using HISAT2. StringTie was used to assemble and quantify transcripts. The DESeq2 package in R (version 3.6.1) was used for measuring differential expression between the conditions. The pheatmap in R was used for clustering. lncRNAs, miRNAs, and mRNAs with a false discovery rate-adjusted *P* < 0.05 and an absolute log_2_FC > 1 were considered as differentially expressed.

### 2.6 Reverse transcription quantitative PCR (rT-qPCR)

Total RNAs were extracted as described above and treated with DNase I (Ambion) to avoid any DNA contamination. The integrity and quality of the RNA were checked by agarose gel electrophoresis. cDNAs were generated using the Superscript II reverse transcriptase (Invitrogen) and random hexamers from 1 μg of total RNA according to the manufacturer’s instructions. A Bio-Rad Cycler was used to carry out RT-qPCR assays. Each reaction contained gene-specific primers (Supplementary Table 4) and SYBR Green Supermix (Bio-Rad). The relative standard curve method was used to quantify relative gene expression. The RT-qPCR data were normalized according to the amplification signals of *gyrA*.

## 3 Results

### 3.1 Choline enhances the spontaneous DNA transformation of *S. pneumoniae*

**FIGURE 1:**
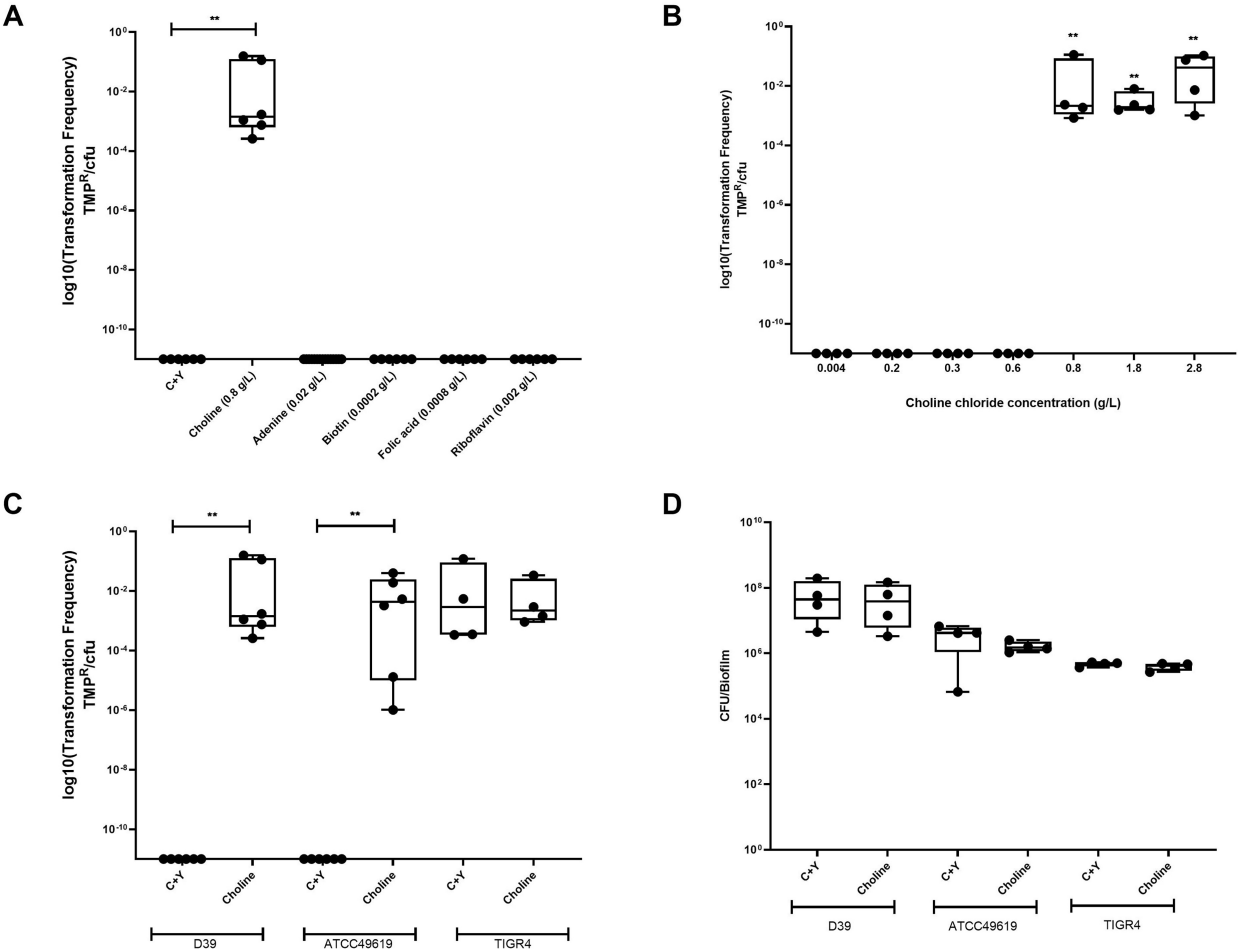
Choline chloride levels modulate natural DNA transformation in *Streptococcus pneumoniae*. Natural transformation (without the addition of CSP) of *S. pneumoniae* D39 occurs with high efficiency in CDM medium but not in C+Y (Peillard-Fiorente et al., 2025). In transformation assays, biofilm-grown bacteria were exposed to a DNA lysate derived from trimethoprim-resistant isogenic cells (D39-DHFR*^I100L^*), and transformation efficiency was calculated as the number of trimethoprim-resistant transformants normalized to the number of viable cells. **(A)** We supplemented the C+Y medium with a selection of 5 chemicals to achieve concentrations similar to what found in CDM (Supplementary Table 1). *S. pneumoniae* D39 cultured as biofilm in C+Y supplemented or not with one of the five selected molecules. Cultures were exposed to a DNA lysate derived from trimethoprim-resistant (D39-DHFR*^I100L^*) isogenic cells. **(B)** Dose-response analysis of choline supplementation on natural transformation of biofilm-grown *S. pneumoniae* D39 in C+Y medium. Transformation efficiency was measured after exposure to D39-DHFR*^I100L^* lysate with increasing concentrations of choline chloride. **(C)** The efficiency of natural transformation of biofilm-grown *S. pneumoniae* D39, TIGR4, ATCC49619 was monitored in C+Y and C+Y supplemented with 0.8 g/L choline chloride after exposure to a D39-DHFR*^I100L^* lysate. **(D)** Biofilm-associated viable cell counts (CFU) of *S. pneumoniae* D39, TIGR4, ATCC49619 grown in C+Y medium in the presence or absence of 0.8 g/L choline chloride. To assess biofilm-associated growth CFU were quantified as described in Materials and Methods. For **(A–D)** each point represents a biological replicate (*n* ≥ 4). Statistical analyses for transformation efficiencies for **(A–C)** were compared using the Kruskal-Wallis test followed by Dunn’s test: **p < 0.01; while for D we used an unpaired *t*-test with Welch’s correction, no significant differences were detected between conditions.

We recently described (Peillard-Fiorente et al., 2025) that *S. pneumoniae* D39, when grown as biofilm and incubated with a D39-DHFR*^I100L^* DNA lysate (DHFR*^I100L^* enables resistance to trimethoprim), could naturally transform in absence of CSP-1 by forming trimethoprim (TMP) resistant colonies when the biofilms were formed in the chemically defined medium CDM (Chao et al., 2017; van de Rijn and Kessler, 1980), but not in the semi-defined medium C+Y (Tomasz and Hotchkiss, 1964). We compared the composition of the media to potentially pinpoint a constituent that could explain this differential natural transformation. Several components differed (Supplementary Table 1) and based on the literature of what is known on DNA transformation, we first selected a series of five chemicals that potentially could impact natural transformation. We selected adenine, biotin, choline, folic acid and riboflavin and adjusted their concentration in C+Y so that it is closer to what found in CDM (Supplementary Table 1). *S. pneumoniae* was seeded in 24-well plates and grown for 72 h in C+Y or C+Y media supplemented with one of those five metabolites. At 24 h the biofilms were incubated with the *S. pneumoniae* D39-DHFR*^I100L^* lysate and natural transformation at an efficiency of 1 × 10^–3^ was observed in the C+Y medium only when supplemented with choline at 0.8 g/L (Figure 1A), a concentration close to what found in CDM (1 g/L). The addition of adenine, riboflavin, folic acid, or biotin had no impact in increasing natural transformation (Figure 1A). A choline titration further revealed a threshold-like response, with transformation detected only at concentration 0.8 g/L and no further increases at higher concentrations (Figure 1B). We have shown previously that the *S. pneumoniae* strain TIGR4, but not the strain ATCC49619 transforms naturally in C+Y medium (Peillard-Fiorente et al., 2025). We tested whether this effect of choline was observed with other *S. pneumoniae* strains. As expected, TIGR4 transformed well in C+Y medium whether choline concentration was adjusted or not (Figure 1C) and interestingly, ATCC49619 could be naturally transformed only when C+Y was supplemented with 0.8 g/L of choline (Figure 1C). Importantly, choline supplementation did not significantly alter biofilm-associated viable cell count (CFU) in D39, ATCC49619, or TIGR4, indicating that the observed increase in transformation is not attributable to changes in biofilm-associated growth (Figure 1D).

While most of our work was carried out with D39 biofilms, we have also showed that choline supplementation in the C+Y medium is associated with natural transformation in planktonic *S. pneumoniae* D39 cells (Supplementary Figure 1).

### 3.2 Choline supplementation modulates gene expression in *S. pneumoniae*

**FIGURE 2:**
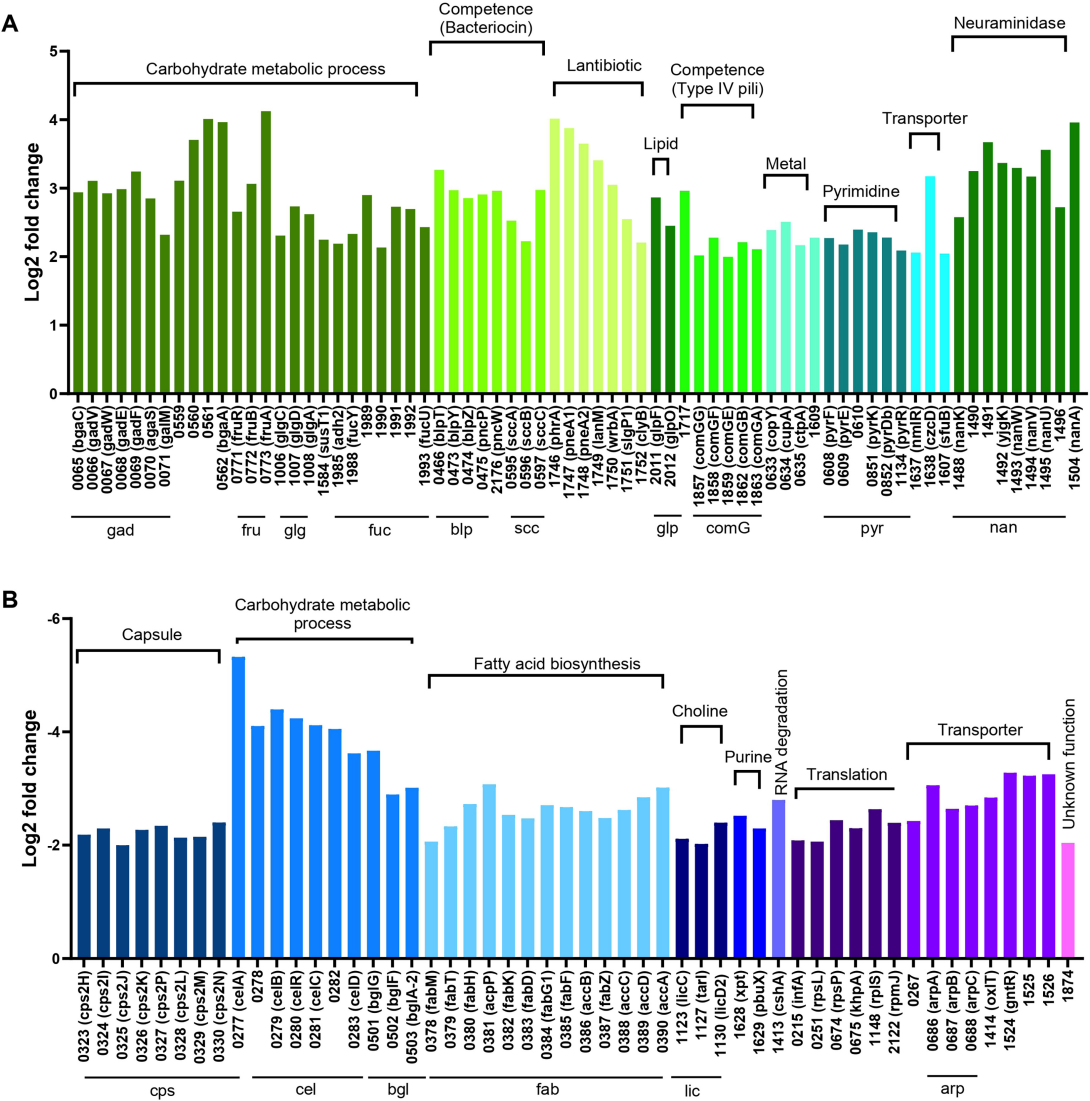
Modulation of operon gene expression in *S. pneumoniae* D39 grown as biofilms in C+Y upon supplementation with choline. Gene expression of *S. pneumoniae* grown as biofilms in C+Y (0.004 g/L of choline) was measured by RNA-seq and compared to cells grown in choline supplemented (0.8 g/L) C+Y medium. The complete gene expression data can be found in Supplementary Table 2. Here is shown a selection of the genes part of operons (underlined below gene numbers- SPV_) whose expression is the most modulated upon the addition of choline. **(A)** Overexpressed operons (log_2_FC ≥ 2); **(B)** downregulated operons (log_2_FC ≤ –2).

To further delve on the role of choline in genetic transformation we compared the transcriptomic response of D39 grown as a biofilm for 72 h in C+Y medium or in C+Y medium supplemented with 0.8 g/L of choline. Cells were harvested and RNA was extracted, processed, and sequenced by next-generation sequencing. The 200-fold increase in choline concentration resulted in marked transcriptomic changes with 356 genes with higher expression [log_2_ fold change (log_2_FC) ≥ 1), Supplementary Table 2] and 267 genes whose expression was downregulated (log_2_FC ≤ −1, Supplementary Table 2) by choline supplementation. We identified several operons showing a notable modulation in their expression, being either upregulated (log_2_FC ≥ 2, Figure 2A) or downregulated (log_2_FC ≤ −2, Figure 2B), in response to choline.

Since choline supplementation led to natural transformation (Figure 1), we investigated whether the expression of genes part of the competence regulon was altered upon choline supplementation. The competence regulon of *S. pneumoniae* induced by the competence stimulating peptide CSP was first established using microarrays (Rimini et al., 2000; Dagkessamanskaia et al., 2004; Peterson et al., 2004) and was recently refined by RNA-seq studies (Slager et al., 2019). This regulon was found to be under the control of the transcriptional regulators ComE, ComX, CiaR, and VraR/HrcA. The expression of several genes part of these regulons was modulated in cells grown in C+Y supplemented with choline (Supplementary Table 3). Half of the 15 operons under the control of ComE, involved in the early phase of competence, were upregulated. This includes two operons for the competence specific sigma factors ComX1 and ComX2 (Lee and Morrison, 1999; Supplementary Tables 2, 3), the ComAB ABC transporter required for CSP export (Hui et al., 1995; Supplementary Tables 2, 3); three operons of the bacteriocin BlpR regulon with roles in neighbor predation (Wholey et al., 2019; Figure 2 and Supplementary Tables 2, 3) and the immunity factor ComM (Straume et al., 2017; Supplementary Tables 2, 3). ComX regulates genes of many operons in later phase of competence (Slager et al., 2019) and some of these operons were upregulated by choline supplementation. This includes the cibABC bacteriocin peptide operon, the late competence DNA transporter ComEC (Silale et al., 2021), the cclA peptidase (Supplementary Tables 2, 3), and the seven genes of the late competence *comG* operon encoding the type IV pilus (Laurenceau et al., 2013; Figure 2A and Supplementary Tables 2, 3). One operon encoding chaperone genes under the control of VraR/HrcA was overexpressed in cells supplemented with choline (Supplementary Table 3). However, none of the CiaR regulated genes were increased in our experiment and in fact some were downregulated (Supplementary Table 3).

The expression of a plethora of genes involved in various metabolic pathways were also modulated upon supplementation with choline (Figure 2 and Supplementary Table 2). For example, a series of genes part of operons involved in carbohydrate metabolism: the *fru, gad*, *glg*, and *fuc* operons for, respectively fructose, galactose, glycogen, and fucose metabolism were upregulated. Notable additional operons whose expressions were upregulated following choline supplementation were implicated in the metabolism of sialic acid, pyrimidine, and the amino acid tryptophan (Supplementary Table 2). In addition to the ComE and ComX induced bacteriocin operons mentioned above, a locus involved in lantibitioc (highly modified peptides part of the bacteriocin family) biosynthesis (SPV_1746-SPV_1753) was also modulated in choline supplemented medium (Figure 2 and Supplementary Table 2).

As somewhat expected, all the eight genes of the *lic* locus (SPV_1123 to SPV_1130) involved in choline metabolism and in teichoic acid precursors were downregulated in cells supplemented with choline (Supplementary Table 2). The *lic* locus includes two operons *lic1* and *lic2* (Johnston et al., 2016) and three genes *licC, tarI*, of the *lic1* operon and *licD2* of the *lic2* operon were decreased by more than (log_2_FC ≤ −2) upon choline supplementation in the C+Y medium (Figure 2B). The capsule of *S. pneumoniae* is known to diminish DNA transformation in *S pneumoniae* (Bradshaw et al., 2020; Li et al., 2016) which is coherent with our observation that the capsule *cps* operon (SPV_0315 to SPV_0331) is downregulated in cells growing in choline-supplemented medium (Supplementary Table 2 and Figure 2B). While, as discussed above, many operons involved in carbohydrate metabolism were upregulated, the *cel* and *bgl* loci, responsible for cellobiose utilization or encoding regulatory proteins for operons coding for genes involved in sugar metabolism were downregulated. Additional operons whose expressions were downregulated following choline supplementation, included operons involved in the biosynthesis of the ATP synthase subunit (SPV_1334-SPV_1341), of purine, of fatty acid (SPV_0378-SPV_0390), and of several operons involved in translation notably ones encoding ribosomal proteins (Supplementary Table 2).

**FIGURE 3:**
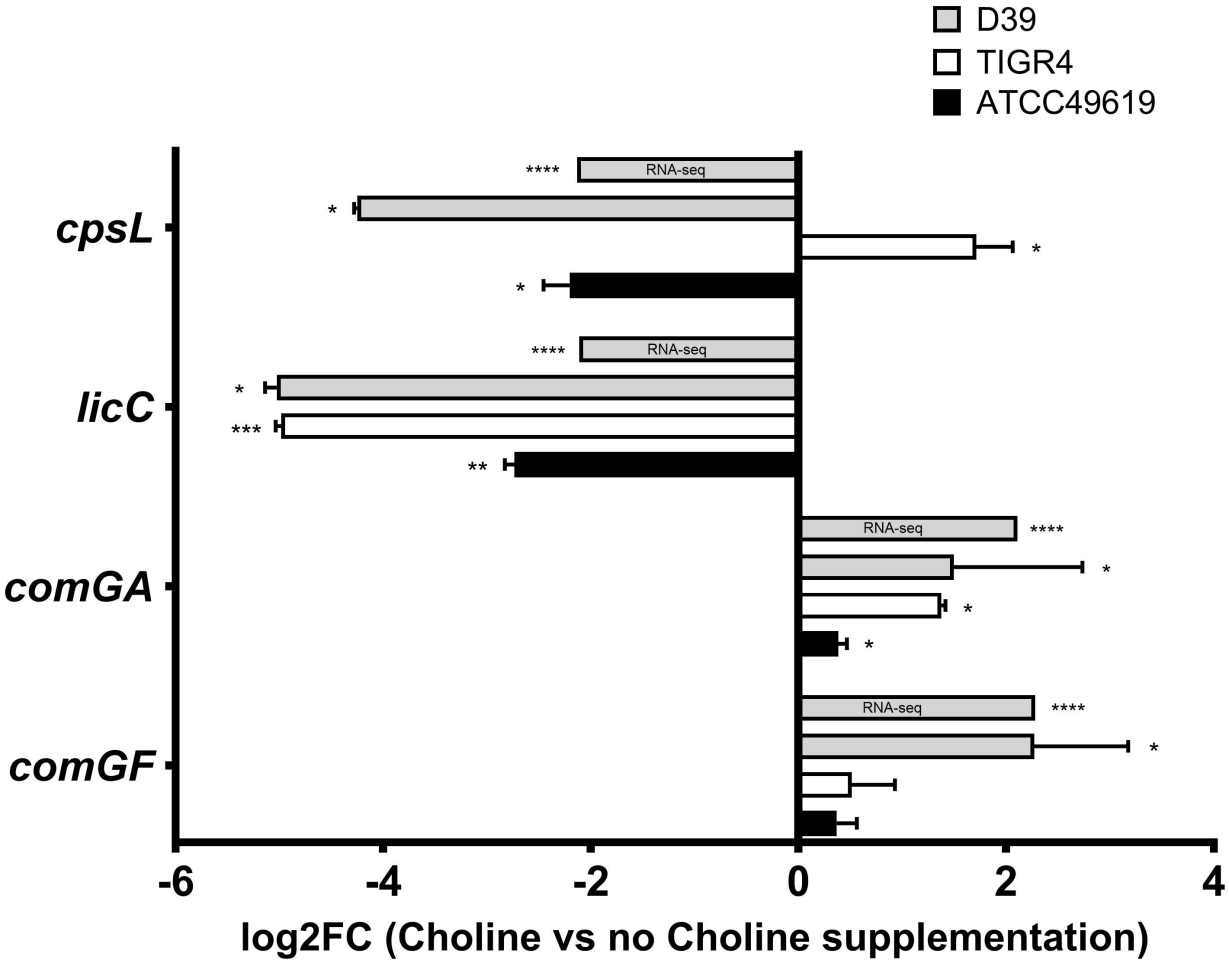
Alterations in gene expression of selected *Streptococcus pneumoniae* genes growing as biofilms upon choline supplementation. The expression of four genes, *cpsL, licC, comGA*, and *comGF* was altered in strain D39 upon supplementation of C+Y medium with 0.8 g/L of choline as measured by RNA-seq (Figure 2 and Supplementary Table 2) and shown here with the bar with the RNA-seq label. The expression of those four genes were monitored by RT-qPCR upon choline supplementation in strains D39, TIGR4, and ATCC49619 encoding, respectively different alleles of *cpsL* (*cps2L, cps4L, rmlA*). RNA levels were normalized based on the amplification signal of *gyrA*. Results are the means of ± SD from three biological replicates. The data for *comGA* and *comGF* for D39 are also found in Supplementary Figure 2. Statistical analyses for the RT-qPCR were carried out using unpaired t-test, with two-tailed *p*-value; *p < 0.05; **p < 0.01; ***p < 0.001. For RNA-seq, significance was assessed using Benjamini-Hochberg adjusted *p*-values (*p*_adj_) calculated by DESeq2; all genes shown reached *p*_adj_ < 0.0001 (****).

The modulated gene expression observed by RNA-seq upon choline supplementation was confirmed by RT-qPCR for a number of genes (Supplementary Figure 2), including the overexpression of genes from the *comG* operon, regulated by ComX (Slager et al., 2019), and the *comA* gene, regulated by ComE (Slager et al., 2019) and detected by RNA-seq (Supplementary Table 3), as well as the downregulation of *celB* (Supplementary Figure 2), part of the *cel* operon (Figure 2B). The down regulation of the *licC* and the *cps2L* genes observed by RNA-seq (Figure 2) were also confirmed by RT-qPCR in D39 growing in C+Y supplemented with 0.8 g/L of choline (Figure 3). Interestingly the expression of the choline metabolic gene *licC* was also downregulated in the TIGR4 and ATCC49619 strains growing in C+Y medium supplemented with 0.8 g/L of choline (Figure 3). Similarly to D39, choline supplementation facilitates natural transformation of ATCC49619 (Figure 1C) and we observed, by RT-qPCR, that its *cps2L* orthologue capsule gene *rmlA* was also downregulated upon choline addition (Figure 3). The *cps4L* gene of TIGR4, however, was not downregulated (Figure 3). The expression of two genes of the pilus encoding operon comG, *comGA*, and *comGF* showed a trend toward modest upregulation in the 3 strains upon choline supplementation (Figure 3).

## 4 Discussion

*S. pneumoniae* D39 biofilms can naturally transform, without exogenous addition of CSP, in CDM medium but not in C+Y (Peillard-Fiorente et al., 2025). By metabolite supplementation of the C+Y medium we found that choline was the main molecule promoting natural DNA transformation of D39 but also of strain ATCC49619 (Figure 1A). A dose-response analysis revealed a threshold-like behavior, with natural transformation becoming detectable only when choline concentrations reached 0.8 g/L or higher (Figure 1B) a concentration similar to what found in the CDM medium (1 g/L) but 200-fold higher than C+Y (0.004 g/L). Choline plays an essential role in the growth of *S. pneumoniae* (Badger, 1944; Rane and Subbarow, 1940) and is known to be important for DNA transformation (Tomasz, 1968). In our assay choline supplementation increases transformation as we provide DNA as DNA lysates, but it has been reported that at high concentration choline hinders the release of choline-binding protein (CBPs), which is crucial in the mechanism of fratricide induced during competence (Steinmoen et al., 2002). While competence-associated DNA uptake and fratricide-mediated DNA release are both part of the same pathway, they are separate processes. The reported effects of choline on CBPs to modulate fratricide do not necessarily limit transformation under conditions where extracellular DNA is available. To understand how choline impacts natural transformation in biofilms, we performed transcriptomic studies and showed that increasing choline concentration had a major impact on gene expression in *S. pneumoniae*. Competence, necessary for DNA transformation, is a dynamic process and our approach of RNA sequencing at one specific point in time, must be considered as a snapshot, yet changes in gene expression were highly informative (Supplementary Tables 2, 3). Serial time points would be of interest to garner further insights on the temporal role of choline, and possibly on the role of other metabolites tested in Figure 1A, in *S. pneumoniae* physiology and in competence for natural transformation.

We observed that choline modulates the expression of many genes and operons under the control of the main transcriptional regulators of the competence regulons (Slager et al., 2019) ComE, ComX, and VraR regulated genes (Supplementary Table 3). This is consistent with the need of *S. pneumoniae* to be in a competent state for effective natural transformation. However, not all genes known to be increased during competence were upregulated during choline supplementation. For example, only half of the operons induced upon CSP addition and regulated by ComE (Slager et al., 2019) were upregulated by choline. It is salient to point out that we analyzed gene expression in biofilms and at a time point (72 h) where we have validated that natural transformation was active in C+Y medium supplemented with choline. Our experimental set up thus differs with studies investigating the kinetics of competence development following CSP addition where gene expression of planktonic cells was studied from 3 to 30 min following CSP addition (Rimini et al., 2000; Dagkessamanskaia et al., 2004; Peterson et al., 2004; Slager et al., 2019). The difference in both the physiological bacterial state (mature biofilm versus planktonic cells) and timing of competence induction are obvious and the choline effects cannot be directly compared to canonical competence but we wanted to highlight that the expression of many genes, as described above and below, are similarly affected by choline and CSP. Gene expression was found to be highly dynamic with early, late and delayed expression of competence genes. When we compared our results with the latest analysis of CSP-induced competence gene expression using RNA-seq (Slager et al., 2019) we noticed that the genes and operons with increased expression in cells grown in C+Y supplemented with choline (ComX1, ComX2, ComAB, BlpR) (Figure 2 and Supplementary Tables 2, 3) overlap with the genes with high expression levels throughout the time course 3–20 min after CSP addition (Slager et al., 2019). This should be interpreted as shared transcriptional features rather than matching temporal dynamics. A similar observation was done with the ComX regulated operons. Indeed, the cibABC, ComEC, cclA, and ComG operons whose expressions were increased after choline supplementation (Supplementary Table 3) where among the operons whose expression was the most upregulated throughout the time course after CSP addition, as determined by RNA-seq (Slager et al., 2019).

There were, however, some ComE or ComX regulated operons that were expressed throughout the full-time course after CSP addition (Slager et al., 2019) that failed to show increased expression following the addition of choline. One of those exception was the ComCDE operon that was not detected in our work. The *comC* gene encodes for a precursor of the CSP peptide that is matured upon its export outside the cell where it can interact with ComD, a histidine kinase part of a two-component system, that will auto phosphorylate and transfer its phosphate to activate the ComE transcription regulator (Salvadori et al., 2019). Choline supplementation, as does CSP, leads to the overexpression of many ComE regulated operons (Supplementary Table 3) and thus choline must have a role in the phosphorylation of ComE. A number of possibilities can be raised. Obviously, our time point (72 h in biofilm) may not have been optimal for detecting *comCDE* overexpression. However, other choline upregulated loci regulated by ComE were observed. Choline may have direct or indirect effects that either increase the affinity of basal amount of CSP and ComD and activate ComE, or may facilitate the phosphorylation of ComE by an alternative kinase. Further work is warranted for determining the detailed mechanism by which choline increases natural competence.

The expressions of other operons, not under the control of the competence transcriptional regulators, were also increased in cells grown with choline supplementation. For example, we found significant overexpression of *nanA* and its operon in response to choline addition (Figure 2). NanA is known for removing sialic acids from the cell surface (Burnaugh et al., 2008) but also for its contribution to biofilm formation (Oggioni et al., 2006; Parker et al., 2009). One potential hypothesis is that its neuraminidase activity may mimic alterations in interactions with host cells. Since host adherence has been linked to competence activation (Minhas et al., 2023), an increased expression of *nanA* by choline could potentially activate competence and contribute to the higher transformation rate observed.

The expression of operons that were downregulated by choline supplementation also provided insights on how choline may increase natural transformation. The *cel* locus genes are downregulated by choline. Since *celR*, the exclusive transcriptional activator of the *cel* locus (Shafeeq et al., 2011), is downregulated this obviously results in decreased *cel* gene locus expression. The link between choline supplementation and *celR* downregulation is unclear but choline changes the expression of several carbohydrate metabolic pathways (Figure 2) and transcriptional activity at the level of the *cel* locus was found to be modulated depending on the carbon source present in the medium (Shafeeq et al., 2011). Our analysis indicated that the expression of at least five operons coding for ribosomal proteins were downregulated (Figure 2 and Supplementary Table 2). This is consistent with expression analysis of CSP treated cells where multiple ribosomal protein genes and full operons were also found to be downregulated (Rimini et al., 2000; Peterson et al., 2004), although in a recent study this was not observed at the earliest time point (3 min) after CSP addition (Slager et al., 2019).

Choline supplementation had a major effect on the expression of operons for fatty acid biosynthesis (SPV_0378-SPV_0390) and for the capsule (SPV_0315-SPV_0331) (Figure 2 and Supplementary Table 2). Expression of these operons were not changed following CSP addition (Rimini et al., 2000; Dagkessamanskaia et al., 2004; Peterson et al., 2004; Slager et al., 2019). Choline is a key constituent of the pneumococcus cell wall and alterations in phospholipid composition may affect *S. pneumoniae* membrane homeostasis and affect competence and DNA transformation. The marked reduction in the expression of the whole capsule operons is even more revealing. Indeed, loss of the capsule in isolates with a mutation in the *cpsE* gene was associated with increased transformability (Schaffner et al., 2014). Of note, the expression of one capsule gene (*rmlA* coding for CpsL) in ATCC49619 was also downregulated, similarly to the *cps2L* D39 gene, upon choline supplementation (Figure 3). This was not observed for TIGR4 but it is salient to point out that TIGR4 is capable of natural transformation in C+Y (Figure 1C) while D39 and ATCC49619 are incapable, except if choline is added (Figure 1). It is likely that many changes are necessary for facilitating natural transformation and since choline facilitates this in D39 and ATCC49619, it is reasonable to posit that they share expression features after choline supplementation that are possibly not necessary for TIGR4. More recently using isogenic strains it was demonstrated that the capsule has an inhibitory effect of transformation of the pneumococcus by interfering with the assembly of the ComG pilus involved in the binding and uptake of the transformed DNA (Ma et al., 2025). In the same study they showed that uncapsulated strain displayed longer pili and here we have shown that choline supplementation both reduce the expression of the capsule and increase the expression of the *comG* operon.

We observed downregulation of the two *lic* operons *lic1* and *lic 2* upon choline supplementation (Supplementary Table 2 and Figure 2B). While this could be expected, there is contrasting result on the expression of the lic operons and choline starvation. One study provided evidence that only the promoter of the *lic1* operon respond to choline starvation in C+Y medium (Johnston et al., 2016) while in another study it was the *lic2* operon that was induced in response to choline deprivation (Desai et al., 2003). In this study we have shown that choline supplementation in C+Y medium decreases the expression of both *lic1* and *lic2* operon, suggesting that these loci respond to choline availability, although supplementation does not mirror the effects of choline deprivation. Of note, the expression of *licC* was down regulated in the three *S. pneumoniae* strains tested when grown in C+Y supplemented with choline (Figure 3). *S. pneumoniae* is highly dependent on choline (Rane and Subbarow, 1940) and possibly that choline deprivation alters bacterial physiology that may have additional impact on the expression of the lic operons, in contrast with cells growing in excess choline. The *lic1* operon is controlled by two promoters, one of which is under the control of the two-component system CiaRH (Johnston et al., 2016; Hakenbeck et al., 1999), well-known to be induced during competence for DNA transformation (Dagkessamanskaia et al., 2004; Peterson et al., 2004). We have shown here that choline supplementation supports DNA transformation (Figure 1) and that it reduces the expression of the genes of the lic 1 operon (Supplementary Tables 2, 3 and Figure 2B) While mechanistically this has yet to be explained, it was observed that while CiaRH is induced upon competence, the lic1 promoter is not (Johnston et al., 2016). None of the known operons regulated by CiaR were shown to be upregulated in cells grown in C+Y supplemented with choline. Consistent with this observation, the CSP mediated fold change expression of genes under the control of CiaR were found in general to be much lower in comparison to genes under the control of ComE or ComX (Slager et al., 2019).

Choline is essential for *S. pneumoniae* growth. We have shown in this study that high concentration of choline, as found in the CDM medium, can facilitate natural transformation independent of the addition of the CSP peptide. Choline modulates the expression of many genes and operons many of which have been associated with DNA transformation. This suggests that choline can contribute to a physiological state permissive for DNA uptake and recombination hence contributing to the evolutionary adaption of the pneumococcus.

## Data Availability

The sequencing reads can be found at the Sequence Reads Archive under BioProject accession PRJNA1285368, with sample accessions SAMN49762523 and SAMN49762524 for *S. pneumoniae* D39 biofilms grown in C+Y with a choline chloride concentration of 0.8 g/L, and sample accessions SAMN49762527 and SAMN49762528 for those grown with a choline chloride concentration of 0.004 g/L.
