# Supplementary material for: On the role of choline in natural DNA transformation in Streptococcus pneumoniae
Source: Front Microbiol. 2026 Jul 9;17:1823130. doi: 10.3389/fmicb.2026.1823130 (PMC13393172; doi:10.3389/fmicb.2026.1823130)
Supplement: Supplementary file 1 [file Table_1.docx]

**Table S1. Composition of chemically defined media: CDM and C+Y**

|  | Synthetic medium | Semi synthetic medium |
| --- | --- | --- |
|  | CDM | C+Y |
| Glucose | 10 g/L | 2.039 g/L |
| Sucrose |  | 0.255 g/L |
| Sodium acetate 3H_2_O (CH_3_COONa) | 4.5 g/L |  |
| Sodium acetate Anhydrous |  | 1.21 g/L |
| Sodium phosphate monobasic (NaH_2_PO_4._H_2_O) | 3.195 g/L |  |
| Sodium phosphate dibasic, anhydrous (NaH_2_PO_4_) | 7.35 g/L |  |
| Potassium phosphate monobasic (KH_2_PO_4_) | 1 g/L | 0.2175 g/L |
| Potassium phosphate dibasic (K_2_HPO_4_) | 0.2 g/L | 4.94 g/L |
| Magnesium sulfate 7xH_2_O (MgSO_4._7H_2_O) | 0.7 g/L |  |
| Magnesium chloride hexahydrate (MgCl_2_·6H_2_O) |  | 0.5098 g/L |
| Manganese sulfate anhydrous (MnSO_4_·H2O) | 0.005 g/L |  |
| Manganese sulfate tetrahydrate (MnSO_4_·4H_2_O) |  | 0.00001723 g/L |
| Ferrous sulfate 7xH_2_O (FeSO_4_·7H_2_O) | 0.005 g/L | 0.0004 g/L |
| Ferric nitrate 9xH_2_O (Fe(NO_3_)_3_·9H_2_O) | 0.001 g/L |  |
| CuSO_4_·5H_2_O |  | 0.0004 g/L |
| ZnSO_4_·7H_2_O |  | 0.0004 g/L |
| MnCl_2_·4H_2_O |  | 0.00016 g/L |
| Calcium chloride anhydrous | 0.005 g/L | 0.0003552 g/L |
| DL-alanine | 0.1 g/L |  |
| L-arginine | 0.1 g/L |  |
| L-aspartic acid | 0.1 g/L |  |
| L-glutamic acid | 0.1 g/L |  |
| Glycine | 0.1 g/L |  |
| L-histidine | 0.1 g/L |  |
| Hydroxy-L-proline (trans-4-hydroxy-L-proline) | 0.1 g/L |  |
| L-isoleucine | 0.1 g/L |  |
| L-leucine | 0.1 g/L |  |
| L-lysine | 0.1 g/L |  |
| L-methionine | 0.1 g/L |  |
| L-phenylalanine | 0.1 g/L |  |
| L-proline | 0.1 g/L |  |
| L-serine | 0.1 g/L |  |
| L-tryptophane | 0.1 g/L | 0.004 g/L |
| L-tyrosine | 0.1 g/L |  |
| L-valine | 0.1 g/L |  |
| L-cysteine | 0.5 g/L | 0.04 g/L |
| L-cysteine HCl | 0.8 g/L |  |
| L-glutamine | 0.2 g/L | 0.2 g/L |
| L-threonine | 0.2 g/L |  |
| Asparagine |  | 0.04 g/L |
| **Adenine *** | 0.02 g/L |  |
| Guanine HCl | 0.02 g/L |  |
| Uracil | 0.02 g/L |  |
| Adenosine |  | 0.0102 g/L |
| Uridine |  | 0.0102 g/L |
| PABA | 0.0002g/L |  |
| **Biotin (D-biotin)** | 0.0002g/L | 0.00000048 g/L |
| **Folic acid** | 0.0008g/L |  |
| Niacinamide | 0.01 g/L |  |
| Nicotinic acid |  | 0.00048 g/L |
| B-NAD | 0.0025 g/L |  |
| D-Ca pantothenate | 0.002 g/L | 0.00192 g/L |
| Pyridoxal HCl | 0.001 g/L |  |
| Pyridoxamine 2HCl | 0.001 g/L |  |
| Pyrodoxine HCl |  | 0.00056 g/L |
| Pyruvate |  | 0.2 g/L |
| **Riboflavin** | 0.002 g/L | 0.000224 g/L |
| Thiamine HCl | 0.001 g/L | 0.000512 g/L |
| Cyanocobalamin | 0.0001 g/L |  |
| Sodium bicarbonate | 5 g/L |  |
| **Choline chloride** | 1 g/L | 0.004 g/L |
| Casamino acids (Difco, vitamin-free, acid hydrolysed) |  | 4 g/L |
| Yeast extract |  | 0.9 g/L |

* molecules in bold were selected for transformation assays in Fig. 1.
