# Supplementary material for: On the role of choline in natural DNA transformation in Streptococcus pneumoniae
Source: Front Microbiol. 2026 Jul 9;17:1823130. doi: 10.3389/fmicb.2026.1823130 (PMC13393172; doi:10.3389/fmicb.2026.1823130)
Supplement: Supplementary file 3 [file Table_3.docx]

**Table S3. Competence related genes differently expressed in sessile cells grown in C+Y with choline (0.8 g/L)**

| **Regulon** | **Gene number** | **Gene name** | **Gene function** | **Biofilm**  **Log2FC (padj < 0.05)** |
| --- | --- | --- | --- | --- |
| **CiaR-regulated genes** | SPV_0868 | prsA/ppmA | Putative parvulin type peptidyl-prolyl isomerase | -1.01 |
|  | SPV_0913 |  | Extracellular protein | -1.56 |
|  | SPV_1123 | licC | Cholinephosphate cytidylyltransferase | -2.11 |
|  | SPV_1124 | licB | Choline permease | -1.84 |
|  | SPV_1125 | licA | Choline kinase | -1.85 |
|  | SPV_1126 | tarJ | Ribulose-5-phosphate reductase | -1.92 |
|  | SPV_1127 | tarI | Ribitol-5-Phosphate cytidylyltransferase | -2.02 |
| **ComE-regulated genes** | SPV_0014 | comX1 | Competence-specific sigma factor | 1.64 |
|  | SPV_0024 | purA | Adenylosuccinate synthetase | -1.10 |
|  | SPV_0049 | comA | Competence-stimulating peptide ABC transporter ATP-binding protein | 0.99 |
|  | SPV_0050 | comB | Competence-stimulating peptide ABC transporter permease protein | 1.36 |
|  | SPV_0466 | blpT |  | 3.59 |
|  | SPV_0472 | blpA | Peptide ABC transporter ATP binding/permease protein | 1.35 |
|  | SPV_2176 | pncW |  | 2.96 |
|  | SPV_0473 | blpY | Bacteriocin immunity protein | 2.97 |
|  | SPV_0474 | blpZ | Immunity protein | 2.86 |
|  | SPV_0475 | pncP | Putative protease | 2.91 |
|  | SPV_1744 | comM | Immunity factor | 1.37 |
|  | SPV_1818 | comX2 | Competence-specific sigma factor | 1.62 |
|  | SPV_1984 | ybbK | Putative stomatin/prohibitin-family membrane protease subunit | 1.17 |
|  | SPV_2062 | tRNA-Glu-5 |  | -1.07 |
|  | SPV_2061 | tRNA-Asn-2 |  | -1.07 |
| **comX-regulated genes** | SPV_2109 | cibC | CibAB immunity factor | 1.96 |
|  | SPV_0132 | cibB | Two-peptide bacteriocin peptide | 1.95 |
|  | SPV_0133 | cibA | Two-peptide bacteriocin peptide | 1.85 |
|  | SPV_0844 | comEC | Late competence DNA transporter | 1.51 |
|  | SPV_1593 | cclA | Type IV prepilin peptidase | 1.36 |
|  | SPV_1857 | comGG | Late competence protein | 2.02 |
|  | SPV_1858 | comGF | Late competence protein | 2.28 |
|  | SPV_1859 | comGE | Late competence protein | 2 |
|  | SPV_1860 | comGD | Late competence protein | 1.88 |
|  | SPV_1861 | comGC | Late competence protein | 1.79 |
|  | SPV_1862 | comGB | Late competence protein | 2.21 |
|  | SPV_1863 | comGA | Late competence protein | 2.11 |
|  | SPV_2427 |  | S-adenosylmethionine-dependent methyltransferase | 2.52 |
|  | SPV_2033 | hpf | Ribosome hibernation promotion factor | 1.76 |
| **vraR-HrcA-regulated-genes** | SPV_0458 | hrcA | Heat-inducible transcription repressor | 1.81 |
|  | SPV_0460 | dnaK | Chaperone protein | 1.85 |
|  | SPV_2171 |  | Hypothetical protein | 1.94 |
|  | SPV_0461 | dnaJ | Chaperone protein | 1.43 |
|  |  |  |  |  |
