# Supplementary material for: On the role of choline in natural DNA transformation in Streptococcus pneumoniae
Source: Front Microbiol. 2026 Jul 9;17:1823130. doi: 10.3389/fmicb.2026.1823130 (PMC13393172; doi:10.3389/fmicb.2026.1823130)
Supplement: Supplementary file 4 [file Table_4.docx]

**Table S4. Primers used in this study**

| all strains qPCR primers | | |
| --- | --- | --- |
| Target gene | | 5’-3’ |
| *gyrA* | Fw | CTTGCTGGCCGGTCTTATGA |
|  | Rv | CCCATAGTTGCACGTCCTGT |
| *licC* | Fw | CCAACGAATGGTTCTTGGT |
|  | Rv | TTTTCTGCAGTTGGAGCATC |
| D39 qPCR primers | | |
| Target gene | | 5’-3’ |
| *comGF* | Fw | ATTAGACCGTTCGCAGTTCG |
|  | Rv | GATGTCCTTGCCATCTTGCT |
| *comGA* | Fw | CGGAGAAATTCGTGACAGCG |
|  | Rv | CCTCGGATACTCTTGGCGTG |
| *cps2L* | Fw | TGATGTTGGCGGGTATTAAG |
|  | Rv | TCTGATCCCAAATTCGGAAC |
| *comGB* | Fw | TGCAGACCTACTTGACAGCC |
|  | Rv | CTGCGTCAACTCCATTCCCT |
| *comA* | Fw | GAATTGACTTCGGATGGGGC |
|  | Rv | ATCCAAAATCAAGACCGGCG |
| *celB* | Fw | TTGTGCAGCAGGAATGTCTT |
|  | Rv | GCGATAGCTTTCCCTCCTTC |
| ATCC49619 and TIGR4 qPCR primers | | |
| Target gene | | 5’-3’ |
| *comGF* | Fw | GCCATCGGTAAGTCAAAGTC |
|  | Rv | GAACCAGTTGATTGTCCTCTG |
| *comGA* | Fw | CATATGAGGGTAGGAGACGA |
|  | Rv | CATACCCGCCACAAACTT |
| ATCC49619 qPCR primers | | |
| Target gene | | 5’-3’ |
| *rmlA* | Fw | CGATTCCAAGAGCTTCTTCA |
|  | Rv | TCCCCAATGATAAAGGCTTG |
| TIGR4 qPCR primers | | |
| Target gene | | 5’-3’ |
| *cps4L* | Fw | TTGGCACATACTGGACAAAA |
|  | Rv | CAAGATCATCTCCAACAGCA |
