# Supplementary figures and images for: On the role of choline in natural DNA transformation in Streptococcus pneumoniae
Source: Front Microbiol. 2026 Jul 9;17:1823130. doi: 10.3389/fmicb.2026.1823130 (PMC13393172; doi:10.3389/fmicb.2026.1823130)

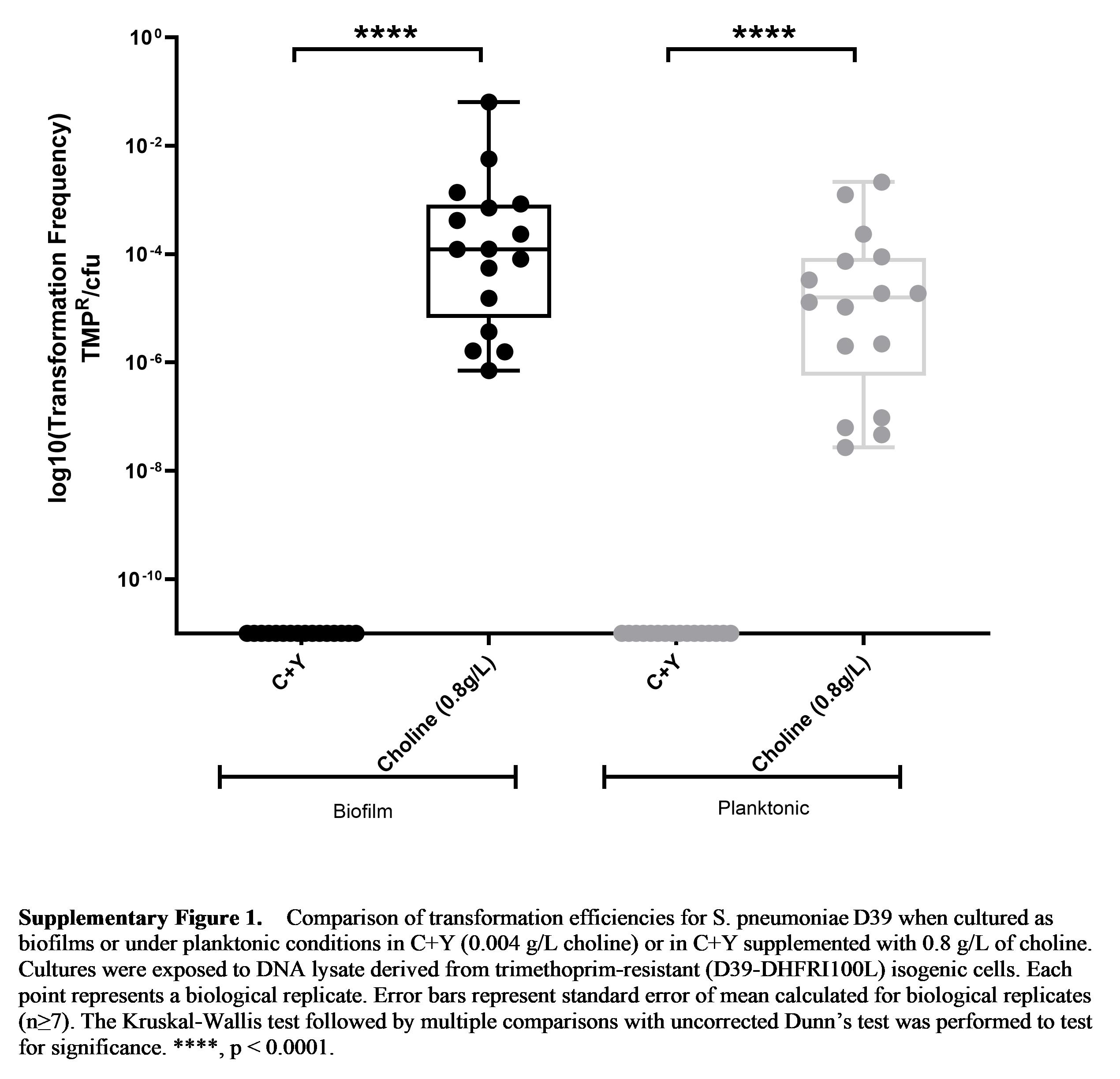

Supplement: Supplementary file 5 [file Supplementary_file_1.jpg]

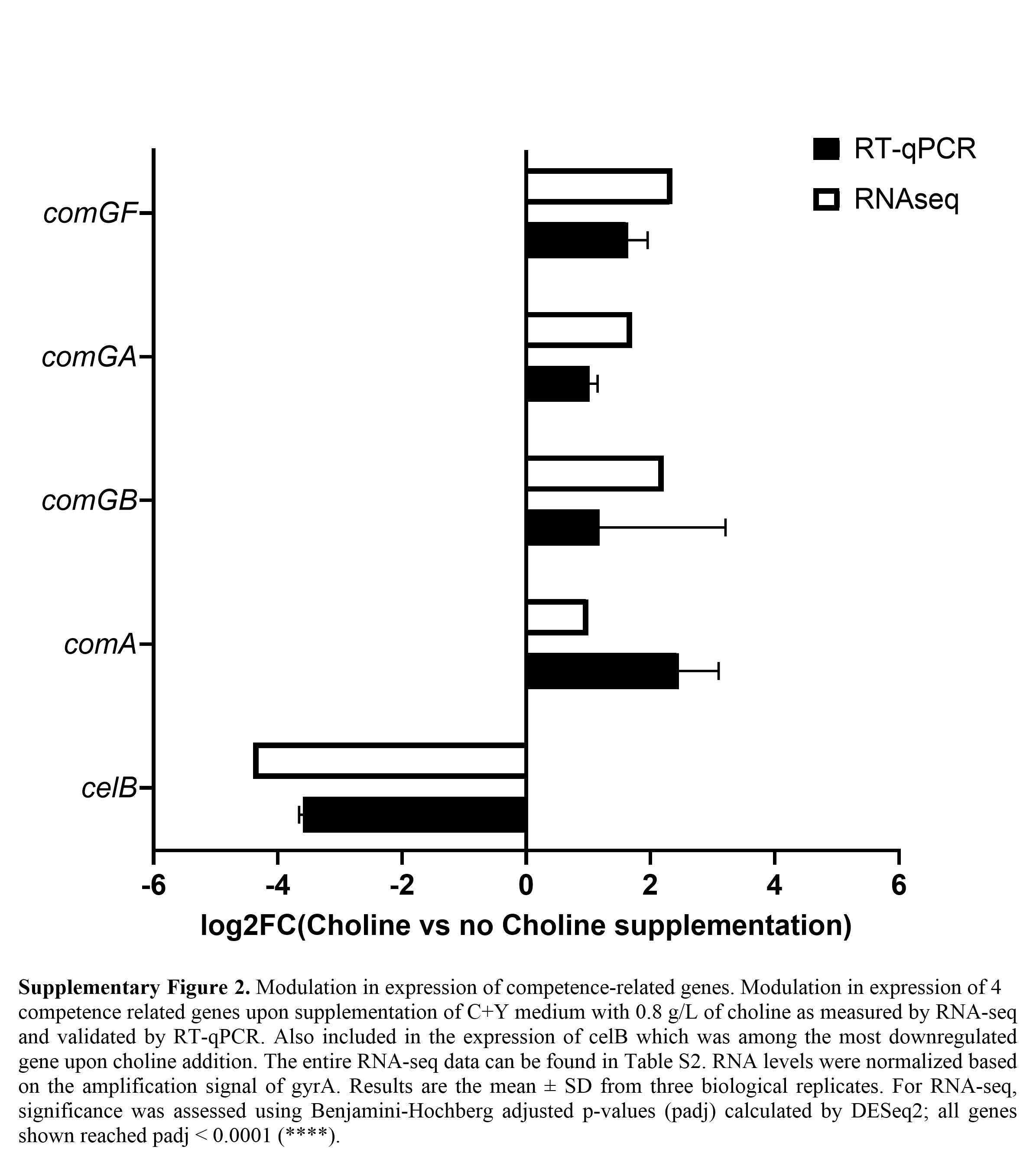

Supplement: Supplementary file 6 [file Supplementary_file_2.jpg]
